# Elucidating the Mechanisms of Chrysanthemum Action on Atopic Dermatitis via Network Pharmacology and Machine Learning

**DOI:** 10.3390/ijms262311262

**Published:** 2025-11-21

**Authors:** Shiying Li, Yongxin Jiang, Chengxiang Hu, Yiyao Ding, Xueqi Fu, Shu Xing, Linlin Zeng

**Affiliations:** Key Laboratory for Molecular Enzymology and Engineering of Ministry of Education, School of Life Sciences, Jilin University, Changchun 130012, China; shiying22@mails.jlu.edu.cn (S.L.); jiangyx23@mails.jlu.edu.cn (Y.J.); hucx23@mails.jlu.edu.cn (C.H.); yiyao25@mails.jlu.edu.cn (Y.D.);

**Keywords:** Chrysanthemum, machine learning, atopic dermatitis, molecular dynamics simulation, network pharmacology

## Abstract

Chrysanthemum (*Chrysanthemum morifolium* Ramat.) has been recognized as both a food and medicinal substance in China since 2002 and possesses antioxidant, anti-inflammatory, antibacterial, and immunomodulatory activities. Previous studies suggest that Chrysanthemum may alleviate skin lesions resembling atopic dermatitis (AD); however, its underlying mechanisms remain unclear. In this study, we integrated network pharmacology and machine learning to systematically explore the potential mechanisms of Chrysanthemum in AD treatment. Four algorithms—Random Forest (RF), Lasso regression with cross-validation (LassoCV), Elastic Net (EN), and Extreme Gradient Boosting (XGB)—were compared, among which the XGB model achieved the best performance (accuracy = 0.9393). Further analysis identified 15 optimal features, two core targets (*PTGS2* and *MMP9*), and one critical pathway (NF-κB signaling). To experimentally validate these findings, HaCaT keratinocytes were co-stimulated with TNF-α and IFN-γ to establish an in vitro inflammatory model, and co-treatment with three major flavonoids from Chrysanthemum—Acacetin, Diosmetin, and Chryseriol—significantly suppressed cytokine-induced COX-2 overexpression and reduced NF-κB p65 phosphorylation, confirming their inhibitory effects on NF-κB activation. These results were consistent with molecular docking and dynamics simulations, which demonstrated that these flavonoids, along with celecoxib, could stably bind to COX-2, thereby enhancing system stability and reducing residue fluctuations at the binding interface, revealing the molecular basis by which Chrysanthemum alleviates AD and supporting its modernization and therapeutic potential.

## 1. Introduction

*Chrysanthemum morifolium* Ramat. (Chrysanthemum) is widely cultivated in Asia and Europe and has a long history of use as both an ornamental and medicinal plant. In China, it is officially recognized as a dual-purpose food and medicine [1]. Among its many varieties, *Chrysanthemum morifolium* Ramat. (Hangju), derived from the dried capitulum, is especially valued for its medicinal efficacy. Modern phytochemical studies have revealed that Chrysanthemum contains diverse active constituents, such as flavonoids, volatile oils, and polysaccharides [2]. These compounds exhibit anti-inflammatory, antioxidant, antibacterial, and immunomodulatory effects. Historically, Chrysanthemum has been used to relieve fever, eye discomfort, and inflammatory conditions, and emerging studies suggest that it may also contribute to alleviating skin inflammation and atopic dermatitis (AD) [3,4]. Nevertheless, its molecular mechanisms remain to be clarified.

AD is a chronic, relapsing inflammatory skin disease associated with genetic predisposition, immune dysregulation, epidermal barrier dysfunction, and environmental triggers [5]. Clinically, AD manifests as pruritus, erythema, exudation, and skin fissuring, predominantly affecting children but also persisting into adulthood [6]. The disease poses a heavy burden on quality of life and public health [7]. Conventional treatment approaches include emollient-based skin care, topical corticosteroids or non-steroidal anti-inflammatory agents, and systemic immunosuppressants or biologics for severe cases [8]. While effective, these strategies are often limited by side effects, cost, and high relapse rates. In addition to these standard therapies, herbal remedies and phytotherapies are increasingly employed as complementary treatments, aiming to reduce inflammation and pruritus with potentially fewer adverse effects. Rich in chemical constituents such as flavonoids, volatile oils, and polysaccharides, Chrysanthemum exhibits anti-inflammatory, antioxidant, and antibacterial effects. It has long been used to mitigate inflammation in AD, with some studies suggesting its potential in alleviating AD symptoms [9,10,11]. However, the precise mechanisms underlying its effects on AD remain elusive and warrant further exploration.

To elucidate the mechanisms through which Chrysanthemum may alleviate AD, integrative computational approaches offer valuable tools [12]. Network pharmacology offers a holistic approach to elucidate the multi-ingredient, multi-target, and multi-pathway nature of traditional herbal remedies, connecting bioactive components to disease-related targets and pathways for identifying pivotal therapeutic modulators [13,14,15]. When integrated with molecular docking, this approach enables precise validation of compound–target binding affinities and specificities, enhancing the reliability of predicted therapeutic mechanisms [16]. Machine learning (ML), as a core branch of artificial intelligence, complements this by efficiently processing high-dimensional biological data, enabling feature selection and enhancing predictive accuracy [17]. Together, network pharmacology and ML synergistically identify core targets and pathways relevant to complex diseases [18]. Molecular dynamics (MD) simulations further strengthen these findings by validating ligand–protein interactions at the atomic level, assessing binding stability and conformational effects [19]. Collectively, this integrative strategy combines predictive capacity with mechanistic validation, providing a robust framework for investigating herbal medicines such as Chrysanthemum [20,21].

In this study, we aim to investigate the therapeutic mechanisms of Chrysanthemum action on AD using a combination of network pharmacology, machine learning, and molecular dynamics simulations. First, network pharmacology was applied to predict potential targets and pathways. Second, gene expression data from healthy individuals and AD patients were analyzed using multiple ML algorithms to identify key features, which were integrated with network pharmacology results. Finally, MD simulations were conducted to validate the binding interactions between Chrysanthemum flavonoids, positive control drugs, and AD-related targets. Through these steps, this study seeks to provide a comprehensive understanding of Chrysanthemum’s molecular basis in alleviating AD and to offer insights for the modernization of herbal medicine and the development of novel therapeutics.

## 2. Results

### 2.1. Analysis of Network Pharmacology

**Figure 1:**
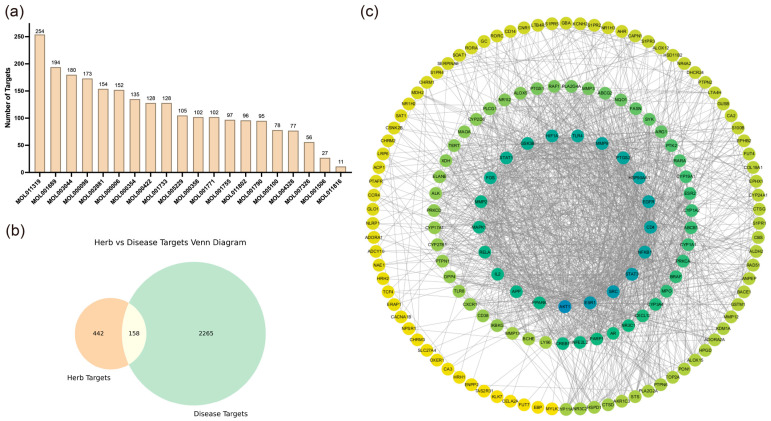
Screening of active compounds from Chrysanthemum and intersection analysis with atopic dermatitis-related targets. (**a**) Bar chart illustrating the number of active small molecules from Chrysanthemum screened in TCMSP and their corresponding target counts. (**b**) Venn diagram depicting the overlap between targets corresponding to small molecules in Chrysanthemum and targets associated with atopic dermatitis. (**c**) Protein–protein interaction (PPI) network constructed from common targets shared by Chrysanthemum and atopic dermatitis.

We collected 20 active compounds from Chrysanthemum using the TCMSP database and predicted a total of 600 corresponding targets through SwissTargetPrediction, SEA, and SuperPred websites, as shown in Figure 1a. Subsequently, 2423 AD-related targets were retrieved from the GeneCards database. The intersection of Chrysanthemum and AD-related targets was obtained and visualized in a Venn diagram, as depicted in Figure 1b.

Next, a protein–protein interaction (PPI) network was constructed using the shared targets, with results shown in Figure 1c. The degree value represents the connectivity of each node with others in the network, with higher degrees indicating greater connectivity. Using CytoHubba, we selected the top 10 molecules with the highest degree values as key targets, as presented in Table S1.

To identify the critical pathways involved in the action of Chrysanthemum, Gene Ontology (GO) and Kyoto Encyclopedia of Genes and Genomes (KEGG) analyses were performed using the shared targets, with results shown in Figures S1 and S2. GO analysis revealed that processes such as response to peptide hormone, regulation of inflammatory response, response to xenobiotic stimulus, intracellular receptor signaling pathway, and steroid metabolic process play critical roles in the anti-inflammatory effects of Chrysanthemum. Additionally, combined with PPI results, KEGG analysis indicated that Chrysanthemum primarily acts on two types of pathways: inflammation-related pathways, where targets such as *AKT1*, *EGFR*, *PTGS2*, and *SRC* not only play central roles in the PPI network but also appear frequently across multiple pathways, including the NF-κB signaling pathway and IL-17 signaling pathway; and immune-related pathways, involving targets such as *CD4* and *MMP9*, as well as pathways like the Chemokine signaling pathway and PD-L1 expression and PD-1 checkpoint pathway in cancer. Given the significant anti-inflammatory effects of Chrysanthemum, we selected AKT1, EGFR, PTGS2, and SRC as key targets.

Subsequently, the 20 active compounds from Chrysanthemum were subjected to batch docking with the aforementioned four targets using ConPlex, with results shown in Table 1. The docking results indicated that the top-ranking compounds were flavonoids from Chrysanthemum. Since top-ranking compounds such as quercetin have been extensively studied for their anti-inflammatory effects, we ultimately selected Acacetin (MOL001689), Diosmetin (MOL002881), and Chryseriol (MOL003044) for further investigation.

### 2.2. Screening of Machine Learning

#### 2.2.1. Preliminary Screening

**Figure 2:**
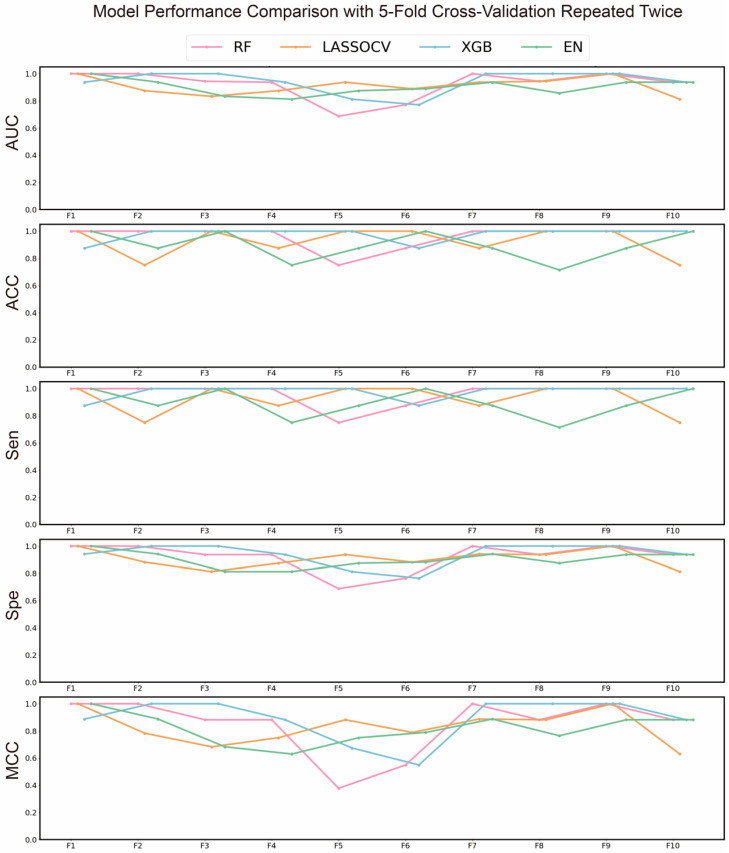
AUC, ACC, MCC, Sen and Spe of each algorithm in each fold of the five-fold cross-validation repeated twice.

**Figure 3:**
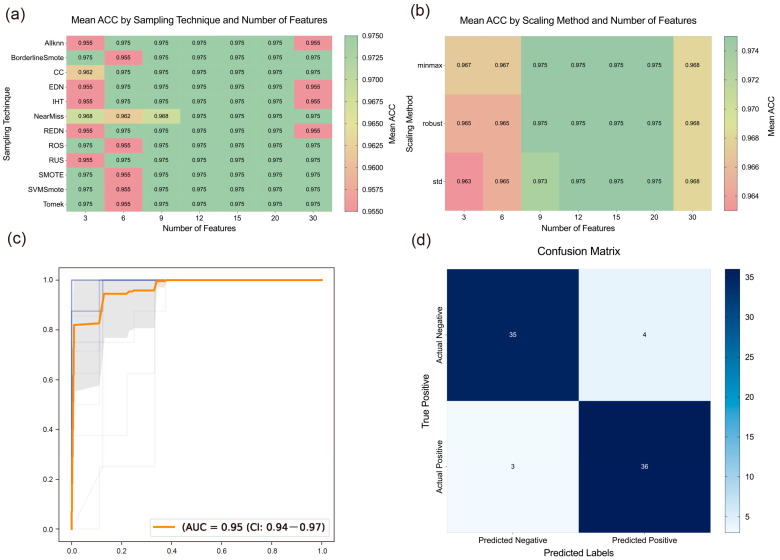
The results of machine learning. (**a**) Heatmap of ACC for the algorithms using different sampling techniques and feature numbers. (**b**) Heatmap of ACC for the algorithms using different scaling methods and feature numbers. (**c**) AUROC curves of the algorithms when applying RFE. (**d**) Confusion matrices of the best model.

To identify the most effective machine learning algorithm for subsequent advanced screening and to preliminarily screen key gene targets associated with Chrysanthemum in combating AD, we evaluated multiple models. The results of the preliminary screening are shown in Figure 2, where the XGBoost (XGB) algorithm demonstrated the best performance, with average AUC, ACC, and MCC values of 0.9396, 0.9393, and 0.8875, respectively. Consequently, the XGB algorithm was selected for subsequent screening. Additionally, recursive feature elimination (RFE) was used to further narrow down the targets, with their corresponding coefficients presented in Table S2. The AUROC curve during the RFE process is shown in Figure 3c.

#### 2.2.2. Further Screening

To further reduce the number and scope of targets, we constructed and evaluated models by combining different sampling methods, scaling methods, and feature quantities. As shown in Figure 3a,b, sampling and scaling methods had a slight impact on results when the feature quantity was either too small or too large. However, when the feature quantity ranged from 12 to 20, their impact became negligible, suggesting that exploring the optimal feature quantity is likely more critical.

To determine the optimal number of features, we analyzed the impact of varying feature quantities on model performance, as shown in Figure 3a,b. The results indicated that model performance deteriorated with either too few or too many genes. This may be due to insufficient gene numbers failing to capture all disease characteristics, while excessive gene numbers introduce redundant information, thus affecting model accuracy. Consequently, we selected the results corresponding to 15 features, achieving an average ACC of 0.975. The top 15 genes identified as critical were *SAMSN1*, *RP2*, *WIF1*, *PTGS2*, *FCGR2A*, *MMP2*, *SOAT1*, *FOS*, *TOP2A*, *TNFAIP8*, *SOX9*, *NR3C2*, *ZBTB10*, *MMP9*, and *TLR4*.

Next, we evaluated the best model derived from the above combinations, with its confusion matrix shown in Figure 3d, demonstrating strong discriminative ability. By integrating these results with the PPI and enrichment analyses, we found that genes such as *PTGS2* and *MMP9* appeared repeatedly, suggesting they may be key targets for Chrysanthemum in combating AD.

### 2.3. Analysis of Molecular Dynamics Simulation

#### 2.3.1. Molecular Docking

**Figure 4:**
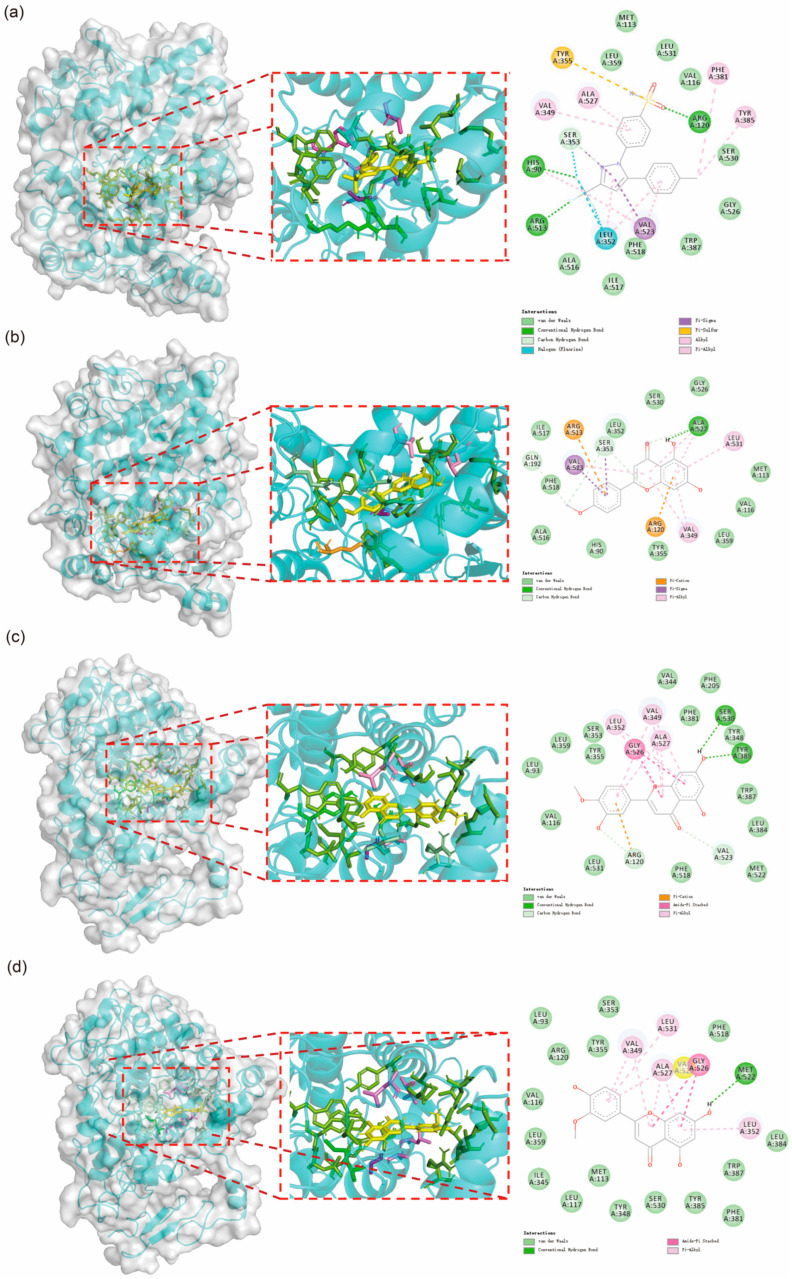
Molecular docking results of the four molecules with COX2. (**a**) Docking results of Celebrex with COX2. (**b**) Docking results of Acacetin with COX2. (**c**) Interaction analysis between Diosmetin and COX2. (**d**) Interaction analysis between Chryseriol and COX2.

The molecular docking results are presented in Figure 4. The findings indicate that Acacetin exhibits a similar interaction pattern with COX-2 as Celebrex, while Diosmetin and Chryseriol share a comparable interaction mode. The interactions between these small molecules and COX-2 primarily involve hydrogen bonds, Pi-Alkyl interactions, carbon-hydrogen bonds, and van der Waals forces. Notably, residues VAL349 and ALA527 consistently form Pi-Alkyl interactions with the small molecules. Additionally, although most residues do not form fixed interactions, residues such as His90, Met113, Val116, Arg120, Leu352, Ser353, Tyr355, Leu359, Tyr385, Ala516, Phe518, Gly526, Ala527, Ser530, and Leu531 frequently appear, underscoring their critical role in the interactions between small molecules and the protein.

#### 2.3.2. Structural Stability Analysis

**Figure 5:**
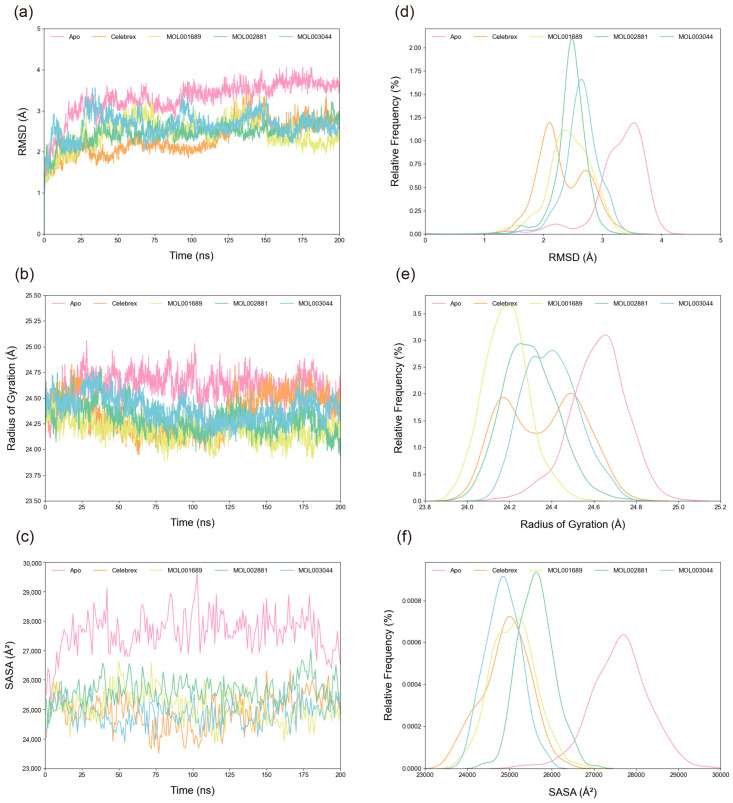
Analysis of structural stability. (**a**) The temporal evolution of the RMSD from their initial structure of the Apo, Celebrex, Acacetin (MOL001689), Diosmetin (MOL002881) and Chryseriol (MOL003044) systems. (**b**) The temporal evolution of the R_g_ from their initial structure of the Apo, Celebrex, Acacetin, Diosmetin and Chryseriol systems. (**c**) The temporal evolution of the SASA from their initial structure of the Apo, Celebrex, Acacetin, Diosmetin and Chryseriol systems. (**d**) Distribution of RMSD values in the four systems. (**e**) Distribution of R_g_ values in the four systems. (**f**) Distribution of SASA values in the four systems. The median (the horizontal line in the center), the mean (the black dot), and the interquartile range (the upper and lower edges of the box) are shown in the box plot for each set of data.

The root mean square deviation (RMSD) of Cα atoms was calculated to assess system stability. As shown in Figure 5a,d, all systems stabilized after 150 ns. The average RMSD values for the Apo, Celebrex, Acacetin, Diosmetin, and Chryseriol systems were 3.29 Å, 2.32 Å, 2.44 Å, 2.46 Å, and 2.65 Å, respectively, indicating enhanced stability upon peptide binding, with the Celebrex system exhibiting the highest stability. Radius of gyration (R_g_) analysis (Figure 5b,e) revealed the compactness of the structures. The average R_g_ values for the Apo, Celebrex, Acacetin, Diosmetin, and Chryseriol systems were 24.62 Å, 24.36 Å, 24.18 Å, 24.30 Å, and 24.40 Å, respectively, consistent with the RMSD results, though the Acacetin system showed a slightly more compact structure. Similarly, solvent-accessible surface area (SASA) trends (Figure 5c,f) indicated average values of 27,617.32818 Å^2^, 24,918.37792 Å^2^, 25,091.92741 Å^2^, 25,627.97598 Å^2^, and 24,856.62225 Å^2^ for the Apo, Celebrex, Acacetin, Diosmetin, and Chryseriol systems, respectively, with the Apo system showing the largest fluctuations and the Chryseriol system the smallest.

**Figure 6:**
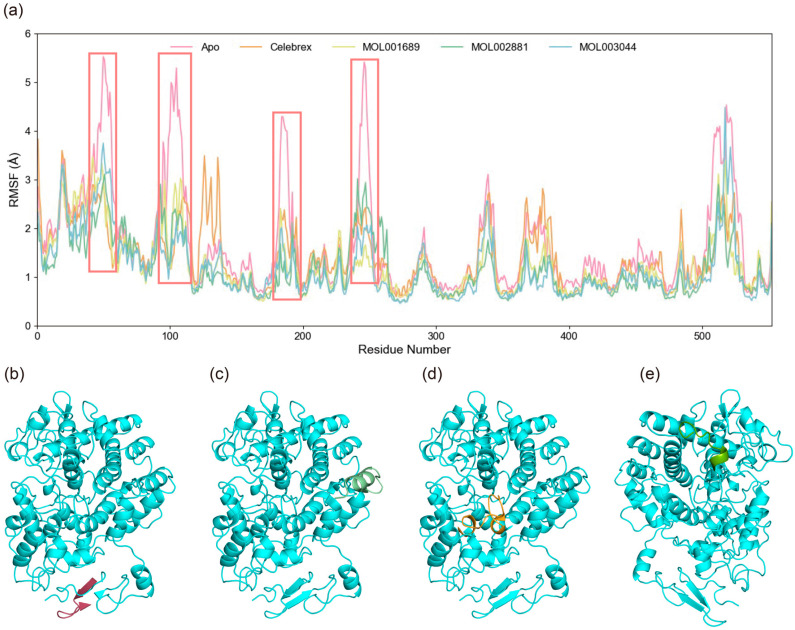
Flexibility analysis and structures of key regions from molecular dynamics simulations of protein–ligand complexes. (**a**) RMSF diagrams for the four systems. The red boxes highlight regions exhibiting markedly increased RMSF values, specifically residues 46–56, 95–110, 241–250, and 505–523, indicating substantial conformational flexibility within these segments of the protein. (**b**) Structure of residues 46–56 in red. (**c**) Structure of residues 95–110 in light green. (**d**) Structure of residues 241–250 in orange. (**e**) Structure of residues 505–523 in green.

The root mean square fluctuation (RMSF) of Cα atoms was calculated to evaluate the specific flexibility of residues in the simulation systems. The average RMSF values for the Apo, Celebrex, Acacetin, Diosmetin, and Chryseriol systems were 1.59 Å, 1.38 Å, 1.27 Å, 1.17 Å, and approximately 1.20 Å, respectively, as shown in Figure 6. In the Apo system, elevated RMSF values were observed at residues 46–56, 95–110, 241–250, and 505–523, indicating significant conformational flexibility in these regions. Notably, these fluctuations were significantly reduced upon small molecule binding, suggesting that interactions with small molecules restricted the dynamic motion of these segments. Conversely, compared to the Apo state, increased RMSF values were observed near the catalytic core residue Tyr385 in the Celebrex and Acacetin systems. This enhanced flexibility in these systems suggests potential structural rearrangements in these regions, which may be critical for mediating the inhibitory effects of small molecules on COX-2. The comparison of RMSF profiles between bound and unbound states highlights localized changes in protein dynamics induced by small molecule binding, potentially linking these residue-specific movements to the functional regulation of COX-2 activity.

#### 2.3.3. MM/PBSA Analysis

**Figure 7:**
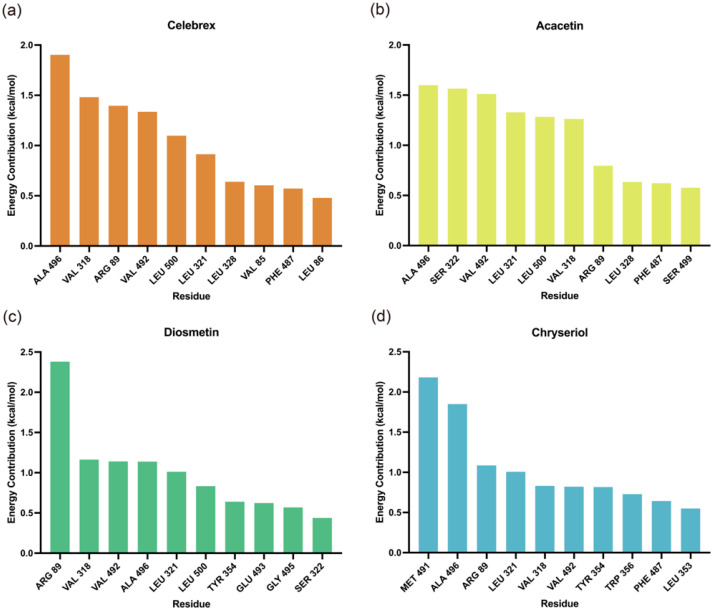
Contributions of amino acid residues to binding free energy in the (**a**) Celebrex, (**b**) Acacetin, (**c**) Diosmetin and (**d**) Chryseriol systems.

Molecular Mechanics/Poisson-Boltzmann Surface Area (MM/PBSA) analysis was performed to identify key residues contributing to the binding of small molecules to COX-2. As shown in Figure 7, residues Arg89, Leu321, Val318, Phe487, Val492, and Leu500 were identified as critical contributors to the binding energy of the complexes. Additionally, the binding free energy values summarized in Table 2 indicate that Acacetin exhibited the highest stability, followed by Celebrex, both demonstrating significantly stronger binding affinities, suggesting greater stability in the COX-2 binding pocket. In contrast, Diosmetin and Chryseriol were relatively less stable, possibly due to the absence of ΔE_ele_ contributions.

#### 2.3.4. Dynamic Cross-Correlation Matrix (DCCM) Analysis

**Figure 8:**
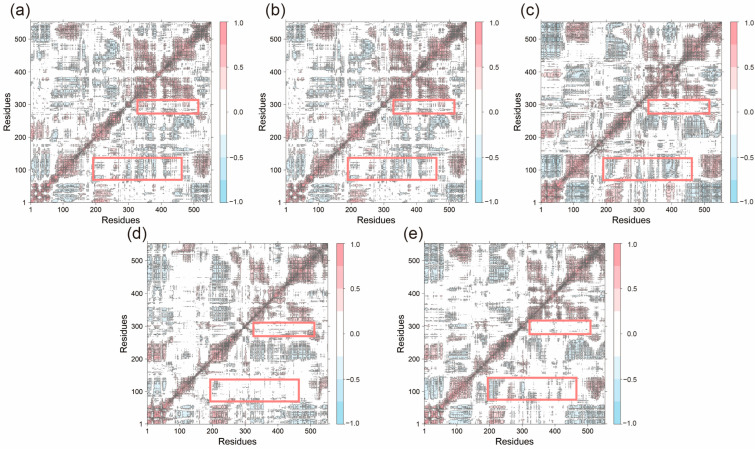
The dynamical cross-correlation matrix diagrams of (**a**) Apo, (**b**) Celebrex, (**c**) Acacetin, (**d**) Diosmetin and (**e**) Chryseriol systems. (The red box encompasses the regions defined by residues 280–300 and 320–520, as well as those defined by residues 70–140 and 200–480).

To further investigate the impact of peptide binding on residue motion dynamics, dynamic cross-correlation matrices (DCCM) were generated for all systems (Figure 8). In most systems, residues 1–75 and 190–250 exhibited significant anti-correlated motions with other residues (indicated by blue regions), reflecting the overall dynamic flexibility of the structure. These anti-correlations were partially reduced in the Acacetin, Diosmetin, and Chryseriol-bound systems. Additionally, positively correlated motions between residues 280–300 and residues around 230–520 were weakened in these systems, highlighting the impact of small molecule binding on the overall system dynamics. Notably, the DCCM profiles of the Apo and Celebrex systems were highly similar, with no substantial differences compared to other systems, suggesting that ligand binding did not significantly disrupt the interactions within the original system.

#### 2.3.5. Secondary Structure Analysis

**Figure 9:**
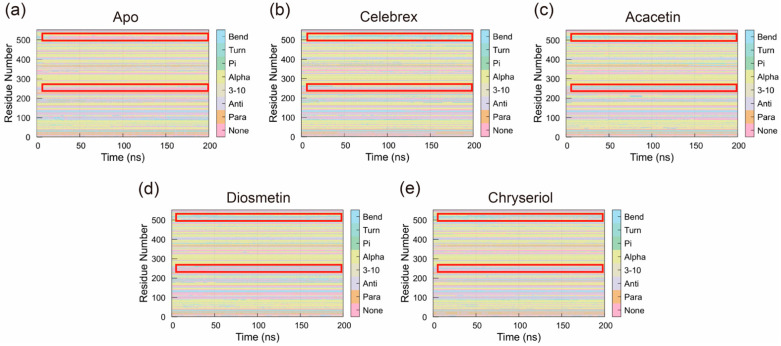
The secondary structure distribution (DSSP) of (**a**) Apo, (**b**) Celebrex, (**c**) Acacetin, (**d**) Diosmetin and (**e**) Chryseriol systems. The red boxed regions correspond to residues 240–270 and 495–540, which exhibit increased Bend and Alpha-helix conformations upon small-molecule binding.

The Definition of Secondary Structure of Proteins (DSSP) was used to analyze changes in the secondary structure of COX-2 during the simulation. As shown in Figure 9, binding of small molecules altered the structural dynamics of COX-2. Specifically, compared to the Apo system, the overall abundance of Bend and Alpha-helix conformations increased in systems bound to small molecules. In the Apo system, regions of residues 240–270 and 495–540, which predominantly lacked specific secondary structures, exhibited increased Bend and Alpha-helix structures upon small molecule binding. These regions showed interconversion between Bend and Alpha-helix structures, with one structure predominating at most times. This suggests that small molecule binding stabilized these regions, maintaining a single Bend or Alpha-helix conformation throughout the simulation. These structural changes likely play a critical role in the mechanism by which small molecules exert their effects on COX-2.

### 2.4. Effects of Acacetin, Diosmetin, and Chryseriol on TNF-α/IFN-γ-Induced COX-2 Expression and NF-κB Activation in HaCaT Keratinocytes

Co-stimulation of HaCaT keratinocytes with TNF-α and IFN-γ is a well-established method for constructing an in vitro skin inflammation model [22]. To evaluate the potential anti-inflammatory effects of the flavonoids Acacetin, Diosmetin, and Chryseriol, we examined their impact on COX-2 expression and NF-κB signaling activation in TNF-α/IFN-γ-stimulated HaCaT cells. The concentration ranges of the three flavonoids were selected based on previous studies [23,24,25].

**Figure 10:**
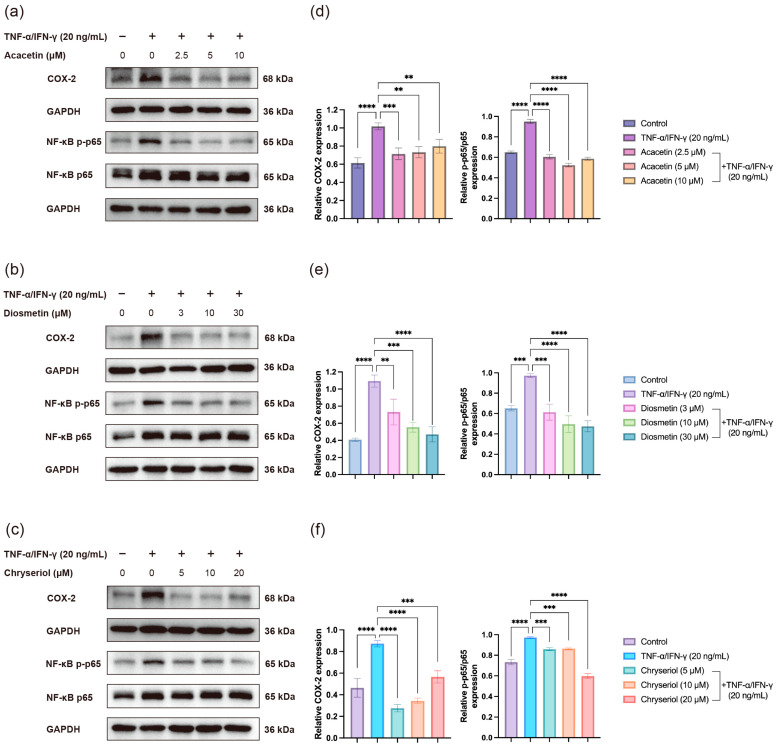
Effects of three flavonoids on TNF-α/IFN-γ-induced COX-2 expression and NF-κB signaling activation in HaCaT cells. HaCaT cells were co-treated with TNF-α and IFN-γ (20 ng/mL each) together with different concentrations of (**a**,**d**) Acacetin (2.5, 5, and 10 μM), (**b**,**e**) Diosmetin (3, 10, and 30 μM), or (**c**,**f**) Chryseriol (5, 10, and 20 μM) for 24 h in three independent experiments. Protein expression levels of COX-2, NF-κB p65, and phospho-NF-κB p-p65 were analyzed by Western blotting, with GAPDH as the loading control. Data are presented as mean ± SD (n = 3). Statistical significance was evaluated using one-way ANOVA followed by Tukey’s multiple comparisons test (GraphPad Prism 9). Significance levels: ** *p* < 0.01; *** *p* < 0.001; **** *p* < 0.0001.

As shown in Figure 10, co-treatment with Acacetin (2.5–10 μM), Diosmetin (3–30 μM), or Chryseriol (5–20 μM) markedly suppressed TNF-α/IFN-γ-induced COX-2 overexpression. Consistently, the phosphorylation level of NF-κB p65, the major subunit responsible for transcriptional activation, was significantly reduced by each compound. These results indicate that all three flavonoids can attenuate NF-κB pathway activation and thereby inhibit inflammatory responses in keratinocytes. The observed molecular changes are in good agreement with our molecular docking and dynamics simulation results, which predicted strong binding affinities of these flavonoids to key components of the NF-κB signaling cascade.

## 3. Discussion

This study leverages an integrative approach combining network pharmacology and machine learning to elucidate the potential mechanisms by which Chrysanthemum alleviates atopic dermatitis (AD). The findings highlight the superior performance of the Extreme Gradient Boosting (XGB) model, which achieved an accuracy of 0.9393, outperforming Random Forest (RF), Lasso regression with cross-validation (LassoCV), and Elastic Net (EN). This high accuracy underscores the robustness of XGB in identifying key molecular features and pathways involved in Chrysanthemum’s therapeutic effects, offering a reliable framework for future studies exploring traditional Chinese medicine (TCM) mechanisms.

The identification of 15 optimal features, with PTGS2 (encoding COX-2) and MMP9 as core targets, provides critical insights into the molecular basis of Chrysanthemum’s efficacy in AD treatment. PTGS2, a key enzyme in prostaglandin synthesis, is well-documented for its role in inflammation, a hallmark of AD [26]. The study’s molecular dynamics simulations further demonstrate that Chrysanthemum-derived flavonoids—Acacetin, Chryseriol, and Diosmetin—exhibit stable binding to COX-2, comparable to the classical COX-2 inhibitor celecoxib. This suggests that these flavonoids may exert anti-inflammatory effects by inhibiting COX-2 activity, thereby reducing prostaglandin-mediated inflammation in AD-like skin lesions [27]. The stable binding and reduced residue fluctuations at the COX-2 interface indicate strong molecular interactions, which could enhance the therapeutic potential of these compounds.

Moreover, the identification of the NF-κB signaling pathway as a critical mechanism aligns with its known role in regulating inflammatory and immune responses in AD [28]. NF-κB activation is associated with the expression of pro-inflammatory cytokines, which exacerbate skin inflammation and barrier dysfunction in AD [29]. By targeting this pathway, Chrysanthemum may modulate the inflammatory cascade, further supporting its therapeutic relevance. The convergence of network pharmacology and machine learning in pinpointing this pathway highlights the power of integrative approaches in dissecting complex TCM mechanisms.

While these findings provide compelling evidence for Chrysanthemum’s role in the treatment of atopic dermatitis (AD), several limitations merit consideration. Firstly, the study relies on computational models and simulations, which, despite their robustness, necessitate validation through in vitro and in vivo experiments to confirm the biological activity of the identified flavonoids and their effects on PTGS2, MMP9, and NF-κB signaling. Secondly, although flavonoids represent the primary active constituents of Chrysanthemum, other bioactive compounds may also contribute to its therapeutic effects. Future research should investigate the synergistic interactions among Chrysanthemum’s diverse phytochemicals to comprehensively elucidate its mechanism of action.

The integration of network pharmacology and machine learning in this study sets a precedent for modernizing TCM research. By identifying specific molecular targets and pathways, this approach not only enhances our understanding of Chrysanthemum’s therapeutic potential but also provides a blueprint for developing novel AD treatments. The flavonoids Acacetin, Chryseriol, and Diosmetin, with their COX-2 inhibitory potential, represent promising candidates for drug development. Further studies should focus on optimizing these compounds for clinical use, evaluating their safety, efficacy, and pharmacokinetic profiles in preclinical and clinical settings.

In conclusion, this study reveals that Chrysanthemum alleviates AD through the inhibition of COX-2 and modulation of the NF-κB signaling pathway, driven by key flavonoids. These findings bridge traditional knowledge with modern scientific approaches, offering valuable insights for the development of TCM-based therapeutics for AD and potentially other inflammatory skin disorders.

## 4. Materials and Methods

### 4.1. Network Pharmacology

#### 4.1.1. Data Preparation

The active small molecules of Chrysanthemum morifolium were sourced from the TCMSP database (https://www.tcmsp-e.com, accessed on 14 May 2025, Beijing Proteomics Research Center, Beijing Institute of Lifeomics, Beijing, China) [30]. Screening criteria included oral bioavailability (OB) greater than 30% and drug-likeness (DL) greater than 0.18. Subsequently, potential targets of these small molecules were predicted using SwissTargetPrediction (http://swisstargetprediction.ch, accessed on 14 May 2025, SIB Swiss Institute of Bioinformatics, Lausanne, Switzerland), SEA (https://sea.bkslab.org, accessed on 14 May 2025, Shoichet Laboratory, University of California, San Francisco, San Francisco, CA, USA), and SuperPred (https://bio.tools/superpred, accessed on 14 May 2025, Charité—Universitätsmedizin Berlin, Berlin, Germany) websites, with targets exhibiting a probability greater than 80% selected as relevant [31,32]. Targets associated with atopic dermatitis were obtained from the GeneCards database (https://www.genecards.org/, accessed on 14 May 2025, Crown Human Genome Center, Weizmann Institute of Science, Rehovot, Israel) [33].

#### 4.1.2. PPI Network and Enrichment Analysis

The intersection of Chrysanthemum-related targets and atopic dermatitis-related targets was determined, and a Venn diagram was constructed. The shared targets were imported into the STRING database (https://www.genecards.org/, accessed on 15 May 2025, STRING Consortium, Lausanne, Switzerland) to build a protein–protein interaction (PPI) network, which was further visualized using Cytoscape 3.10.3 (Cytoscape Consortium, La Jolla, CA, USA) [34,35]. The CytoHubba plugin (National Tsing Hua University, Hsinchu, Taiwan) was employed to rank targets based on degree values, identifying key targets [36]. The shared targets were subjected to Gene Ontology (GO) and Kyoto Encyclopedia of Genes and Genomes (KEGG) pathway enrichment analyses using Rstudio (version 2024.12.1+563, Posit Software, PBC, Boston, MA, USA), with further analysis incorporating the identified key targets [37,38,39,40]. Molecular docking was performed using ConPlex between key targets and all small molecules, and three small molecules were selected as key molecules for subsequent analyses [41].

### 4.2. Machine Learning

#### 4.2.1. Data Collection

The dataset for machine learning was derived from the Gene Expression Omnibus (GEO) database (https://www.ncbi.nlm.nih.gov/geo/, accessed on 28 July 2025, National Center for Biotechnology Information, National Institutes of Health, Bethesda, MD, USA). Gene expression data from healthy individuals and patients with atopic dermatitis were collected from GSE232127, GSE174582, and GSE286907 [42,43,44]. The intersection of genes from these datasets was used to construct a new dataset comprising all genes, and the expression matrices from the three datasets were merged to form the comprehensive machine learning dataset.

#### 4.2.2. Primary Screening

Feature selection was conducted using Random Forest (RF), Lasso regression CV(LassoCV), Elastic Net (EN), and Extreme Gradient Boosting (XGB) algorithms, with the merged expression matrix as the input dataset. For RF, an ExtraTreesClassifier with 500 estimators and a random state of 42 was used to ensure reproducibility. For Lasso, LassoCV was applied with a random state of 42 and 50 parallel jobs. For EN, ElasticNet was used with 5-fold cross-validation, a maximum of 10,000 iterations, α values ranging from 0.001 to 10, and l1_ratio values ranging from 0.1 to 0.9. For XGB, an XGBClassifier with 300 estimators, a learning rate of 1.0, a maximum depth of 3, and a random state of 42 was employed.

Five-fold cross-validation was performed, repeated twice. Features selected by these four algorithms were combined with key genes identified from network pharmacology analysis for further screening. Algorithm performance was evaluated using accuracy (ACC), area under the curve (AUC), Matthews correlation coefficient (MCC), sensitivity (Sen), and specificity (Spe). The best-performing algorithm was selected for further analysis.

Recursive Feature Elimination (RFE) was subsequently applied to refine the selected features. The area under the receiver operating characteristic curve (AUROC) was generated for analysis, and genes were ranked by importance to prepare for the next stage of analysis.

#### 4.2.3. Secondary Machine Learning-Based Filtering

Using XGB as the base algorithm, various scaling methods (StandardScaler, QuantileTransformer, PowerTransformer, and RobustScaler) and sampling techniques (Synthetic Minority Oversampling Technique (SMOTE), Adaptive Synthetic Sampling (ADASYN), Random Undersampling (RUS), Random Oversampling (ROS), Borderline-SMOTE (B-SMOTE), Support Vector Machine SMOTE (SVM-SMOTE), Tomek Links, and NearMiss) were combined with multiple features. Five-fold cross-validation was performed, repeated twice, and algorithm performance was assessed using ACC scores. The optimal algorithm was selected, and a confusion matrix was generated. The selected features were comprehensively evaluated and integrated with PPI and pathway enrichment analyses to identify key features.

### 4.3. Molecular Dynamics Simulation

#### 4.3.1. Molecular System Preparation

The crystal structure of the COX-2 protein (PDB ID: 1CX2) was obtained from the Protein Data Bank (https://www.rcsb.org/, accessed on 9 August 2025, Worldwide Protein Data Bank (wwPDB), Rutgers University, Piscataway, NJ, USA) [45,46]. The structure of the positive control drug was sourced from the PubChem database (https://pubchem.ncbi.nlm.nih.gov/, accessed on 9 August 2025, National Center for Biotechnology Information, National Institutes of Health, Bethesda, MD, USA), while the structures of the three small molecules were obtained from the TCMSP database [47]. Prior to molecular docking, the COX-2 structure was preprocessed in Discovery Studio 2021 (Dassault Systèmes BIOVIA, San Diego, CA, USA) by removing water molecules and ligands around the active site.

#### 4.3.2. Molecular Docking Protocol

Molecular docking was performed using AutoDock Vina (version 1.2.0, The Scripps Research Institute, La Jolla, CA, USA) [48]. Docking results were visualized and analyzed using PyMOL 2.1 (Schrödinger, Inc., New York, NY, USA) and Discovery Studio 2021 to determine the optimal binding conformations of small molecules with the protein [49]. The best binding pose was selected as the initial structure for subsequent molecular dynamics (MD) simulations.

#### 4.3.3. Molecular Dynamics Protocol

MD simulations were conducted using AMBER 22 software (University of California, San Francisco, San Francisco, CA, USA). Five simulation systems were constructed: COX-2 protein alone (apo group), COX-2 bound to Celebrex (Celebrex group), COX-2 bound to Acacetin (MOL001689 group), COX-2 bound to Diosmetin (MOL002881 group), and COX-2 bound to Chryseriol (MOL003044 group). The simulations aimed to investigate the interactions and dynamic structural changes between small molecules and COX-2. All simulations utilized the AMBER ff19SB force field [50]. Systems were solvated in the OPC water model and placed in an octahedral periodic box with an 8 Å buffer. Sodium ions (Na^+^) were added to neutralize the charge [51]. Long-range electrostatic interactions were handled using the Particle Mesh Ewald (PME) method with a cutoff distance of 10 Å [52].

Following system construction, initial energy minimization was performed in two stages: 6000 steps of steepest descent followed by 6000 steps of conjugate gradient minimization. The systems were then gradually heated to 310 K using the Nosé-Hoover thermostat under NVT ensemble conditions. After heating, 200 ns equilibration simulations were conducted for each system under NVT conditions with a 2 fs time step. System stability was assessed by monitoring the root mean square deviation (RMSD) of kinetic and potential energy, temperature, and other parameters. Trajectory snapshots were recorded every 0.1 ns, generating 2000 frames per simulation. AmberTools 23 was used for trajectory analysis, calculating parameters such as RMSD, radius of gyration (R_g_), solvent-accessible surface area (SASA), root mean square fluctuation (RMSF), and definition of secondary structure of proteins (DSSP).

#### 4.3.4. Dynamic Cross-Correlation Matrix (DCCM) Analysis Pipeline

DCCM analysis was performed to quantify correlated motions between protein residues and reveal functionally relevant dynamic coupling networks [53]. After removing translational and rotational motions, time-series positional data for all residues were extracted from MD trajectories. Normalized cross-correlation coefficients were calculated for each residue pair (i, j). Positive values indicated correlated motions, while negative values indicated anti-correlated motions. Results were visualized as a heatmap using R Studio (version 2024.12.1+563, Posit Software, PBC, Boston, MA, USA), with color intensity representing correlation strength.

#### 4.3.5. Molecular Mechanics/Poisson-Boltzmann Surface Area (MM/PBSA) Analysis

The MMPBSA method was used to calculate the binding free energy between the protein and ligands from MD trajectories. Binding affinity was evaluated by decomposing contributions from van der Waals, electrostatic, and solvation energies, identifying key residues influencing binding energy.

### 4.4. Cell Culture and Treatment

The immortalized human keratinocyte cell line (HaCaT; Servicebio, Wuhan, China) was cultured in high-glucose Dulbecco’s Modified Eagle Medium (DMEM) supplemented with 10% fetal bovine serum and 1% penicillin–streptomycin. To induce an inflammatory response, cells were stimulated with recombinant human tumor necrosis factor-α (TNF-α) and interferon-γ (IFN-γ) (20 ng/mL each; PeproTech, Cranbury, NJ, USA) for 24 h. For treatment assays, cells were simultaneously stimulated with TNF-α and IFN-γ and co-treated with Acacetin (2.5, 5, or 10 μM), Diosmetin (3, 10, or 30 μM), or Chryseriol (5, 10, or 20 μM) for 24 h. All compounds were purchased from MedChemExpress (MCE, Monmouth Junction, NJ, USA).

### 4.5. Western Blot Analysis

Cells were lysed in RIPA buffer containing protease and phosphatase inhibitors, and total protein concentrations were determined using the BCA assay. Equal amounts of protein (20 μg per lane) were separated by SDS–PAGE and transferred onto polyvinylidene difluoride (PVDF) membranes (Millipore, Burlington, MA, USA). The membranes were blocked with 5% bovine serum albumin (BSA) for 1 h at room temperature and then incubated overnight at 4 °C with primary antibodies against NF-κB p65 (Cell Signaling Technology, Danvers, MA, USA), COX-2, and phosphorylated NF-κB p65 (p-p65) (Proteintech, Wuhan, China). After washing, the membranes were incubated with HRP-conjugated secondary antibodies for 1 h at room temperature.

For sequential detection, the PVDF membrane used for p-p65 detection was stripped using a mild stripping buffer, re-blocked with 5% BSA, and re-incubated overnight with an anti-p65 antibody to assess total p65 levels. Protein bands were visualized using an enhanced chemiluminescence (ECL) detection system and quantified using ImageJ (version 1.53t, National Institutes of Health, Bethesda, MD, USA) software.

## 5. Conclusions

Through an integrated analysis combining network pharmacology and machine learning, we identified PTGS2 and MMP9 as key targets of Chrysanthemum in the treatment of AD, along with critical pathways such as the NF-κB signaling pathway and IL-17 signaling pathway. By conducting 200 ns molecular dynamics simulations on five systems, we elucidated the inhibitory mechanisms of four small molecules on COX-2. Notably, Acacetin exhibited a mechanism of action more similar to Celebrex, suggesting a higher likelihood of selective inhibition of COX-2. Binding of all small molecules to COX-2 enhanced the overall stability of the systems. Furthermore, ligand binding selectively suppressed the motion of flexible regions (e.g., residues 95–110 and 505–523) while increasing flexibility near the catalytic core residue Tyr385, indicating that inhibition is mediated through localized conformational rearrangements. Consistent with these computational predictions, co-treatment of TNF-α/IFN-γ-stimulated HaCaT keratinocytes with Acacetin, Diosmetin, or Chryseriol significantly suppressed COX-2 overexpression and reduced NF-κB p65 phosphorylation, confirming their inhibitory effects on NF-κB pathway activation and inflammatory responses. MM/PBSA analysis revealed that Acacetin exhibited the most favorable binding free energy, surpassing Celebrex, whereas Diosmetin and Chryseriol displayed lower stability, potentially due to the absence of electrostatic contributions (ΔE_ele_). Additionally, secondary structure analysis showed that ligand binding induced the formation of stable α-helix and Bend structures in disordered regions (residues 240–270 and 495–540), reducing conformational transitions and likely enhancing the rigidity of the active site.

Despite these insights, several inherent limitations of the in silico methodology must be acknowledged. First, the study did not incorporate in vivo ADME (absorption, distribution, metabolism, and excretion) profiles or metabolic transformations of the compounds, which may significantly alter their bioavailability, efficacy, and target engagement in biological systems. Second, network pharmacology analyses are inherently static and fail to capture the dynamic, time-dependent regulatory processes that govern gene expression, protein interactions, and signaling cascades in living organisms. Third, while molecular docking and dynamics simulations predict binding affinities and conformational changes, these do not guarantee functional inhibition or therapeutic outcomes in complex cellular or physiological contexts, where off-target effects, feedback loops, and compensatory mechanisms may dominate. These constraints underscore the preliminary nature of our findings and highlight the necessity of future experimental validation through in vitro and in vivo studies.

Collectively, this study elucidates the inhibitory mechanisms of Chrysanthemum against AD, providing a robust theoretical foundation for the development of related food and pharmaceutical products and offering valuable insights for the design of selective COX-2 competitive inhibitors and anti-inflammatory drugs.

## Figures and Tables

**Table 1:** Docking score of active small molecules with key target complexes.

Components	AKT1	EGFR	PTGS2	SRC	Average
MOL000006	−9.64	−7.70	−9.38	−8.55	−8.82
MOL000098	−9.52	−7.56	−9.50	−8.67	−8.81
MOL001689	−9.20	−7.85	−9.50	−8.45	−8.75
MOL003044	−9.08	−7.81	−9.43	−8.48	−8.70
MOL007326	−10.57	−7.95	−7.08	−9.11	−8.67
MOL002881	−9.12	−7.77	−9.10	−8.67	−8.66
MOL000422	−9.06	−7.46	−9.56	−8.40	−8.62
MOL000354	−8.65	−7.67	−9.42	−8.68	−8.60
MOL001790	−10.98	−8.22	−5.32	−9.83	−8.59
MOL001755	−10.90	−8.43	−5.49	−9.09	−8.48
MOL011802	−10.05	−7.72	−5.23	−10.76	−8.44
MOL000358	−10.77	−8.03	−4.72	−9.33	−8.21
MOL001771	−10.50	−8.18	−5.34	−8.53	−8.14
MOL001506	−8.83	−6.98	−8.82	−7.41	−8.01
MOL001733	−8.98	−7.36	−6.35	−8.71	−7.85
MOL004328	−7.94	−6.52	−7.54	−7.10	−7.27
MOL005229	−8.90	−6.74	−4.71	−7.51	−6.97
MOL011319	−6.92	−5.42	−8.13	−7.15	−6.91
MOL011816	−8.12	−6.86	−5.48	−6.76	−6.81
MOL005100	−7.37	−5.98	−5.52	−6.93	−6.45

Docking scores of the indicated active small molecules with AKT1, EGFR, PTGS2, and SRC were calculated using molecular docking simulations. Average values represent the mean binding score across the four targets.

**Table 2:** Comparative MMPBSA Analysis of Ligand–Protein Binding Energetics and Stability.

System	Celebrex	Acacetin	Diosmetin	Chryseriol
ΔE_vdw_	−47.87 ± 0.20	−44.30 ± 0.23	−40.41 ± 0.19	−40.01 ± 0.21
ΔE_ele_	−11.55 ± 0.41	−25.90 ± 0.50	−2.64 ± 0.28	−16.55 ± 0.48
ΔG_solv_	37.58 ± 0.42	44.82 ± 0.58	32.98 ± 0.30	41.75 ± 0.44
ΔG_gas_	−59.42 ± 0.40	−70.20 ± 0.61	−43.05 ± 0.31	−56.56 ± 0.46
ΔG_total_	−21.85 ± 0.26	−25.37 ± 0.24	−10.07 ± 0.30	−14.81 ± 0.26

Binding free energy components (kcal/mol) of ligand–protein complexes were calculated by MM/PBSA, including van der Waals energy (ΔE_vdw_), electrostatic energy (ΔE_ele_), solvation free energy (ΔG_solv_), gas-phase free energy (ΔG_gas_), and total binding free energy (ΔG_total_).

## Data Availability

The original contributions presented in this study are included in the article/Supplementary Materials. Further inquiries can be directed to the corresponding author.

## Supplementary Materials

The following supporting information can be downloaded at https://www.mdpi.com/article/10.3390/ijms262311262/s1.

## Abbreviations

The following abbreviations are used in this manuscript:

AD	atopic dermatitis
RF	Random Forest
LassoCV	Lasso regression with cross-validation
EN	Elastic Net
XGB	Extreme Gradient Boosting
ML	Machine learning
MD	Molecular dynamics
OB	oral bioavailability
DL	drug-likeness
PPI	protein–protein interaction
GO	Gene Ontology
KEGG	Kyoto Encyclopedia of Genes and Genomes
GEO	Gene Expression Omnibus
ACC	accuracy
AUC	area under the curve
MCC	Matthews correlation coefficient
Sen	sensitivity
Spe	specificity
RFE	Recursive Feature Elimination
AUROC	area under the receiver operating characteristic curve
SMOTE	Synthetic Minority Oversampling Technique
ADASYN	Adaptive Synthetic Sampling
RUS	Random Undersampling
ROS	Random Oversampling
B-SMOTE	Borderline-SMOTE
SVM-SMOTE	Support Vector Machine SMOTE
RMSD	root mean square deviation
R_g_	radius of gyration
SASA	solvent-accessible surface area
RMSF	root mean square fluctuation
DSSP	definition of secondary structure of proteins
DCCM	Dynamic Cross-Correlation Matrix
MM/PBSA	Molecular Mechanics/Poisson-Boltzmann Surface Area
