# Supplementary material for: Elucidating the Mechanisms of Chrysanthemum Action on Atopic Dermatitis via Network Pharmacology and Machine Learning
Source: Int J Mol Sci. 2025 Nov 21;26(23):11262. doi: 10.3390/ijms262311262 (PMC12692186; doi:10.3390/ijms262311262)
Supplement: Supplementary file 1 [file ijms-26-11262-s001.zip › ijms-3951430-supplementary.pdf]

Figure S1. Results of GO enrichment analysis.
Figure S2. Results of KEGG enrichment analysis.

Table S1. Top 10 genes ranked by degree in the PPI network.
Table S2. Top 10 targets preliminarily identified by machine learning and their corresponding weight coefficients in the model.

Figure S1

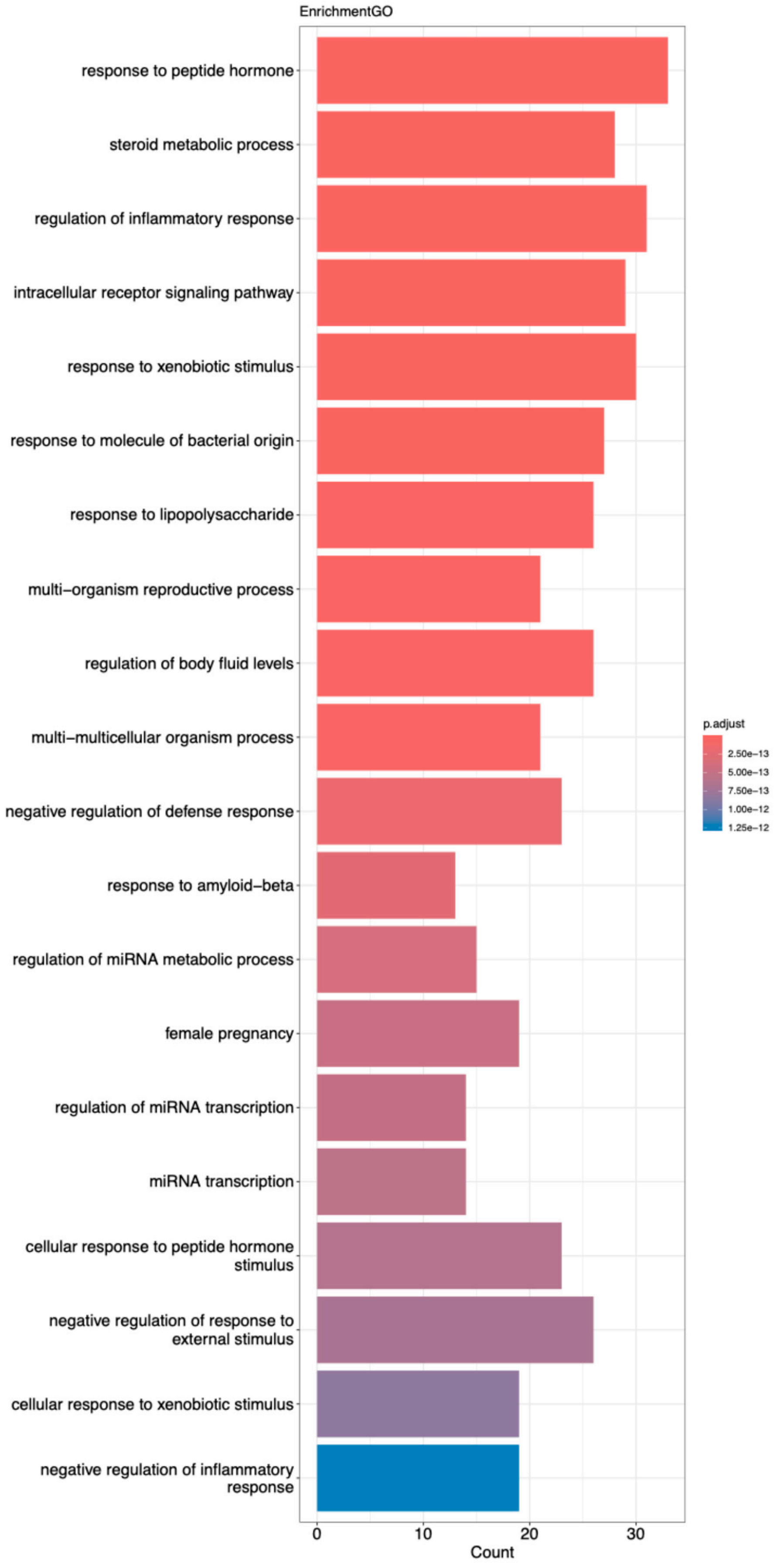

EnrichmentGO
response to peptide hormone
steroid metabolic process
regulation of inflammatory response
intracellular receptor signaling pathway
response to xenobiotic stimulus
response to molecule of bacterial origin
response to lipopolysaccharide
multi−organism reproductive process
regulation of body fluid levels
multi−multicellular organism process
negative regulation of defense response
response to amyloid−beta
regulation of miRNA metabolic process
female pregnancy
regulation of miRNA transcription
miRNA transcription
cellular response to peptide hormone stimulus
negative regulation of response to external stimulus
cellular response to xenobiotic stimulus
negative regulation of inflammatory response
0
10
20
30
Count
p.adjust
2.50e−13
5.00e−13
7.50e−13
1.00e−12
1.25e−12

Figure S2

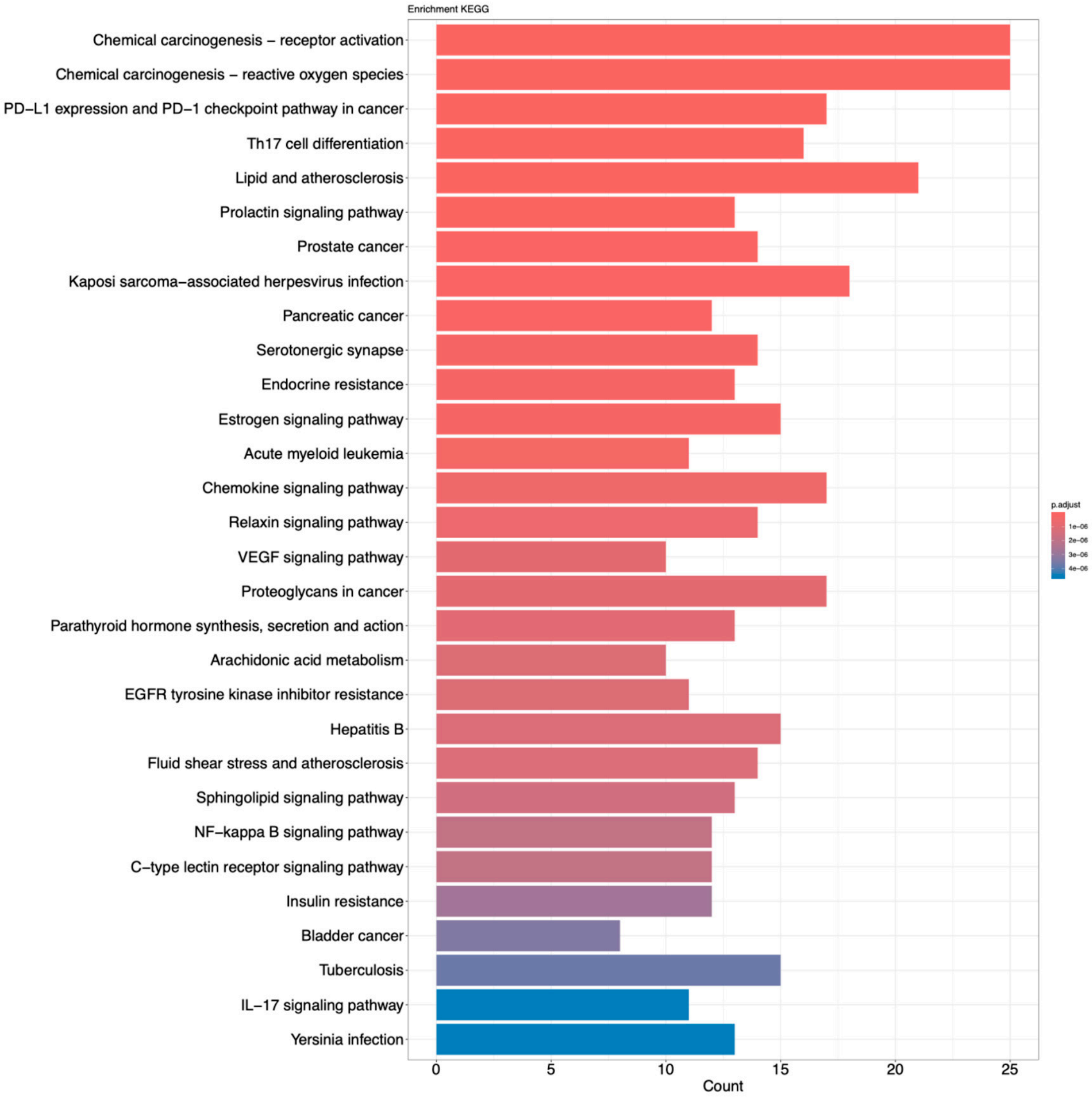
Enrichment KEGG
Chemical carcinogenesis – receptor activation
Chemical carcinogenesis – reactive oxygen species
PD–L1 expression and PD–1 checkpoint pathway in cancer
Th17 cell differentiation
Lipid and atherosclerosis
Prolactin signaling pathway
Prostate cancer
Kaposi sarcoma–associated herpesvirus infection
Pancreatic cancer
Serotonergic synapse
Endocrine resistance
Estrogen signaling pathway
Acute myeloid leukemia
Chemokine signaling pathway
Relaxin signaling pathway
VEGF signaling pathway
Proteoglycans in cancer
Parathyroid hormone synthesis, secretion and action
Arachidonic acid metabolism
EGFR tyrosine kinase inhibitor resistance
Hepatitis B
Fluid shear stress and atherosclerosis
Sphingolipid signaling pathway
NF–kappa B signaling pathway
C–type lectin receptor signaling pathway
Insulin resistance
Bladder cancer
Tuberculosis
IL–17 signaling pathway
Yersinia infection
0
5
10
15
20
25
Count
p.adjust
1e–06
2e–06
3e–06
4e–06

Table S1. Top 10 genes ranked by degree in the PPI network.

Rank	Name	Score
1	AKT1	81
2	ESR1	70
3	SRC	69
4	STAT3	66
5	CD4	60
5	NFKB1	60
5	EGFR	60
8	HSP90AA1	59
9	PTGS2	57
9	MMP9	57

Table S2. Top 10 targets preliminarily identified by machine learning and their corresponding weight coefficients in the model.

Rank	Target	Coefficient
1	SAMSN1	63
2	RP2	39
3	WIF1	37
4	PTGS2	33
5	FCGR2A	31
6	MMP2	30
6	SOAT1	30
8	FOS	27
8	TOP2A	27
10	TNFAIP8	25
